# Monstrosity by Inclusion

**Published:** 1876-01

**Authors:** 


Monstrosity by Inclusion.—At a recent meeting of the Richmond Academy of Medicine (*Virginia Medical Monthly*), Dr. M. M. Walker reported the following case: Last spring he was called upon by a young gentleman who had noticed a slight muco-purulent stain upon his shirt, and complained of an annoying sensation at a point about an inch and a half behind the anus. On examination, the Doctor found two or three small depressions behind the anus, like small-pox pits; but finding no other cause for the irritation, he dismissed the case. Recently the patient returned, having noticed some pus which had been discharged from a fistula at the seat of one of the "pits." The probe was passed about two inches into the fistula, which was found to be closed at its upper limit near the coccyx; there was no evidence that there had ever been any communication whatever with the rectum. Dr. Walker opened the fistula with a bistoury, guided on a grooved director, but saw nothing along the course of the wound worthy of mention. The fistulous track was touched with nitrate of silver, and poultices were ordered to be applied.

After two or three days, Dr. W. noticed a black looking substance protruding from the wound, which, on being pulled out, proved to be a bundle of light-brown, downy hairs—about the size of an ordinary "lock of hair"—discolored at one point by the nitrate silver. Nothing in the history of the case gave any ground for the belief that these hairs had entered from the rectum, while there was no doubt that they had occupied their position as a fœtal development. Acting as a foreign body, however, and the part being, perhaps, irritated by sitting down, or something of the kind, an abscess had been caused, which sought to empty itself at the point indicated. The patient had felt some uneasiness about the locality for the previous two years. The wound of incision healed kindly.

